# Estimating the costs of school closure for mitigating an influenza pandemic

**DOI:** 10.1186/1471-2458-8-135

**Published:** 2008-04-24

**Authors:** Md Z Sadique, Elisabeth J Adams, William J Edmunds

**Affiliations:** 1 Modelling and Economics Unit, Health Protection Agency, 61 Colindale Avenue, London, NW9 5EQ, UK; 2 Economics Department, City University, Northampton Square, London, EC1V 0HB, UK

## Abstract

**Background:**

School closure is a key component of many countries' plans to mitigate the effect of an influenza pandemic. Although a number of studies have suggested that such a policy might reduce the incidence, there are no published studies of the cost of such policies. This study attempts to fill this knowledge gap

**Methods:**

School closure is expected to lead to significant work absenteeism of working parents who are likely to be the main care givers to their dependent children at home. The cost of absenteeism due to school closure is calculated as the paid productivity loss of parental absenteeism during the period of school closure. The cost is estimated from societal perspective using a nationally representative survey.

**Results:**

The results show that overall about 16% of the workforce is likely to be the main caregiver for dependent children and therefore likely to take absenteeism. This rises to 30% in the health and social care sector, as a large proportion of the workforce are women. The estimated costs of school closure are significant, at £0.2 bn – £1.2 bn per week. School closure is likely to significantly exacerbate the pressures on the health system through staff absenteeism.

**Conclusion:**

The estimates of school closure associated absenteeism and the projected cost would be useful for pandemic planning for business continuity, and for cost effectiveness evaluation of different pandemic influenza mitigation strategies.

## Background

Pandemic influenza has been a national and international public health concern for many years. The continuing global spread of the H5N1 strain in birds, and associated human cases has highlighted that a pandemic can occur at any time. As a result many countries have revised and updated their pandemic plans. In such plans the use of non-pharmaceutical interventions (NPI) are proposed to help reduce the number of cases and slow the epidemic spread, particularly if vaccines or antivirals are unavailable or become ineffective because of resistance [1]. School closure is one of the key components of many countries' non-pharmaceutical mitigation strategies [2,3] because of the propensity of influenza epidemics to be amplified in school settings [4]. Although there are a number of studies that have attempted to estimate the possible epidemiological impact of school closure [5-11], there are no published studies of the cost of this strategy. The major concern with closing schools is that it will result in an increase in absenteeism due to childcare needs. This could be expected to have adverse consequences for business continuity and costs to the economy. This paper aims to estimate the economic cost of school closure to the United Kingdom. In addition those sectors likely to incur the greatest costs are identified.

## Methods

The impact of school closure is measured in terms of lost income from missed work of working parents as a consequence of school closure. In this study, the cost is estimated from the societal perspective which calculates the paid productivity loss of parental absenteeism during the period of school closure. The two methods that are most widely used for the valuation of productivity cost are the 'human capital method' (HCM) and the 'friction cost method' [12]. The HCM estimates the value of potential lost production (or income) from a financial point of view [13]. The friction cost method is based on the idea that the amount of production lost as a result of disease (or event, e.g., school closure) is confined to the period needed to replace a sick worker (often assumed to be around three months) [14,15]. Since a pandemic is only expected to last about three months, the friction method and HCM would be expected to give similar results. We have used the HCM here because of the short time horizon [16].

The spring 2005 Labour Force Survey (LFS) weighted regional dataset was used [17] to estimate the absenteeism and its consequent cost. We first estimate the proportion of the workforce that are likely to be the main caregivers and that have dependent children. Dependent children is defined, here, as those under 16 years of age. This definition was chosen as the Labour Force Survey records the living arrangements of adults with children under 16 years. We have therefore estimated the proportion of labour force that are main care givers for the children under 16 years of age in the household (women who are either the head of the household or the spouse of the head of the household, or are cohabiting with the head of the household and who do not have other adults in the household, or men who do not have other adults in the household, but do live with children under the age of 16 years). This gives the number of working parents who are likely to be the main carer for children and may be most liable to take absenteeism to care for these children. These figures are estimated in the following way.

Firstly, we derived the number of parent workers in the population. Three queries of the LFS were done. For the first, the number of individuals were extracted by age group (AGEC: 16–64, 65–99), sex (SEX: male, female), if they reported that they were in paid work in the previous week (WRKING) and if they have one or more dependent children aged under 16 years in the household (FDPCH16), and cross-tabulated by their relationship to the head of the household (RELH96). In the second and third extracts, all variables were retained except age, which was substituted for the industry of employment (INDS92M). From the cross tables of above variables, we have calculated the following information:

(a) Total number of working mothers (single mother or cohabiting with spouse or partner) who are assumed to be the primary caregivers of children. We expect that a major part of absenteeism cost will accrue from these working mothers.

(b) Total number of single/lone fathers who are likely to be absent from work to take care of their child during school closure. The Equal Opportunity Commission [18] reports that the number of families headed by lone fathers in 2005 was 180,000. Given the employment rate of 78%, we get 140,400 working lone fathers.

(c) The above two components give us the number of parent workers who are likely to be absent from work. These estimates are made for the UK workforce as a whole, and by sector excluding those where the information is not known or classified as working outside the UK. The aggregate estimate is also adjusted by the presence of grandparents within the same household. It is assumed that grandparents are most likely to provide alternative care to their grandchild in case of emergency which will allow the respective parents to work.

(d) The absenteeism rate is also calculated in terms of work-days lost per week. This is estimated using the gender-specific employment rate in each industrial sector and patterns of employment (full time and part-time) by gender. In 2005, 39% of women with dependent children and 22% of women without dependent children in the workforce were part-timers, the corresponding proportions for men were 4% and 9% respectively [19].

We then translate the loss of working hours/days per worker into monetary values according to their wages. According to the neoclassical economic model, wage rates equal the value of marginal revenue generated by an additional worker under full employment, and therefore it reflects the value of lost production. We derived the average weekly wage from the sector specific wage rate (variable gross weekly wage (GRSSWKC) by variables SEX, and industry division in the main job (INDS92M)) from Quarterly LFS 2005 April [20] and also used average wage from a Department for Work and Pensions (DWP) survey [21]. We also adjust for the fact that 39% of working mothers are part-timers and the rest work full-time [19]. There is no specific number of hours that makes someone full or part-time, but we assume that a part-timer works half of the hours that a full timer works.

The cost of absenteeism was estimated from two approaches-aggregate and by industrial sector. Firstly, we calculated the cost of absenteeism using aggregate absenteeism figures from LFS and average wage from DWP survey. Alternatively in the industrial sector approach, we estimate the absenteeism for each industrial sector (based on LFS statistics). This industry specific absenteeism was then evaluated in monetary values using both sector specific wage (from LFS) and average wage from DWP. These industry costs were aggregated to give an aggregate national estimate.

The baseline estimates do not allow for any other informal care, apart from that provided by other adults in the household. A report from the Office of National Statistics [22] suggests that overall 54% of working mothers use informal childcare by friends, neighbours, family or childminders for all or part of their childcare. A similar estimate is also reported by the Department for Education and Skills [23]. In our scenario analysis, the cost of absenteeism is adjusted by this figure.

It has been suggested that effective labour-time is reduced less than proportionately from absenteeism as work colleagues may be able provide some cover, and workers may be able to catch-up on some delayed tasks when they return. Although no estimates of this elasticity of production with respect to labour are available for the UK, estimates for the Netherlands suggest a value of 0.8. We have also adjusted our cost estimates by this factor in the scenario analysis. It is also probable that some individuals (particularly in certain sectors) would be able to perform some work whilst caring for their children. We were not able to find an estimate for this parameter. As a proxy we used the figure that 30% of all UK households have access to broadband [24], and assumed, in our scenario analysis that this would reflect the average productivity of workers at home. As a best-case (least cost) scenario we adjusted for the proportion who have access to informal care, the elasticity of production, and the proportion of parents assumed to be able to work from home simultaneously.

We assume in the base-case that, with the exception of lone-fathers, women will take absenteeism to care for children. In the scenario analysis we estimate the cost of school closure if 50% of the absenteeism results from men taking time off to care for their dependent children at home.

Estimates of the cost of school closure are presented in 2005 prices, and are given for the UK as a whole (population ~60 million) and by week. The cost of school closure is assumed to be proportional to the length of school closure.

## Results

**Figure 1:**
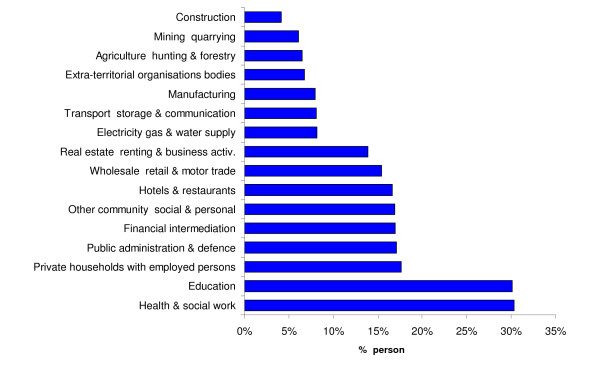
The proportion of the workforce who are likely to be the main caregiver for dependent children in the home, by sector.

An estimated 38% of the workforce has dependent children (aged < 16 years) living within the household. Overall, 15.5% of the workforce is estimated to be comprised of women who have dependent children in the home, and would be expected to provide childcare to their children in the event of school closure. A further 0.6% of the workforce is fathers with dependent children in the household, but with no other adults (lone fathers). Thus the aggregate level of absenteeism due to closing of school is estimated to be 16.1%. The rate of absenteeism in different sectors varies significantly. Figure 1 shows the estimated proportion of the workforce who are likely to be responsible for children <16 years of age by sector (the fishing sector, which accounts for less than 1% of absenteeism is not shown in the graph). The figure clearly shows that the health and social work sector is most likely to be affected by school closure – an estimated 31% of the workforce is responsible for dependent children in the home, roughly twice the national average, as this sector employs a high proportion of women (79%). The educational sector also employs a large proportion of women, and therefore the absenteeism rate would also be very high (~31%). Although presumably the requirements for staff will be reduced if schools are closed.

Absenteeism rates expressed in days (or whole-time equivalents) are lower than that measured in persons (Table 1). This is due to the fact that a greater proportion of women work part-time as compared to their male counterparts. The aggregate absenteeism rate in terms of days is 14.2%. The health sector is still the most affected with around 28% of work-days lost through school closures.

**Table 1:** Estimated proportion of work-days lost through school closure by sector

**Industrial sector**	**Absenteeism rate (in days)**
Agriculture, hunting & forestry	5.6%
Fishing	0.6%
Mining & quarrying	5.3%
Manufacturing	6.9%
Electricity, gas & water supply	7.2%
Construction	3.6%
Wholesale, retail & motor trade	13.8%
Hotels & restaurants	14.9%
Transport storage & communication	7.1%
Financial intermediation	15.1%
Real estate, renting & business activities	12.2%
Public administration & defence	15.2%
Education	27.6%
Health & social work	27.9%
Other community social & personal	15.1%
Private households with employed persons	15.9%
Extra-territorial organisations bodies	5.9%

Without adjusting for informal care, or the elasticity of labour, or the possibility of working from home, then the cost of absenteeism is estimated at close to £1 billion per week of school closure (Table 2). Table 2 represents 3 estimates of the cost of school closure from two main approaches. Column 1 and 2 represent cost estimate from industrial sector specific absenteeism figures, where column 1 quantifies the monetary value of absenteeism in sector specific wage rate specified by LFS and column 2 quantifies in terms of wage specified by DWP. Column 3 represents cost of absenteeism calculated from aggregate data from LFS and aggregate wages as specified in LFS. Although the estimates are similar, they are arrived at by different approaches.

**Table 2:** Weekly cost of school closure (in million £) under a range of different assumptions. LFS represents estimated costs based on the Labour Force Survey and DWP based on Department of Work and Pensions estimates of wages.

	**Sectoral**		**From aggregate figure**
	
	**LFS**	**DWP**	**aggregate adjusted**
Base case	986.0	865.2	865.5
Informal care adjusted	453.6	398.0	398.1
Effective labour adjusted	788.8	692.2	692.4
Work from home adjusted	690.2	605.7	605.8
Effective labour, informal care, and work from home adjusted	254.0	222.9	223.0
Absenteeism of 50% working fathers	1286.0	1128.5	1128.8

The estimated aggregate loss of output (due to work absenteeism) as a % of 2005 GDP for period of school closure ranging from two to twelve weeks is reported in Table 3. It is clear that prolonged school closure can have a significant impact on GDP.

**Table 3:** Range cost of school closure as a % of GDP.

		**Base case**	**Informal care 54%**	**Effective labour 80%**	**Work from home 30%**	**Effective labour, informal care, & work from home adjusted**	**Absenteeism of 50% working fathers**
12 weeks	**LFS**	0.97%	0.44%	0.77%	0.68%	0.25%	1.26%
	**DWP**	0.85%	0.39%	0.68%	0.59%	0.22%	1.10%
8 weeks	**LFS**	0.64%	0.30%	0.51%	0.45%	0.17%	0.84%
	**DWP**	0.56%	0.26%	0.45%	0.40%	0.15%	0.74%
6 weeks	**LFS**	0.48%	0.22%	0.39%	0.34%	0.12%	0.63%
	**DWP**	0.42%	0.19%	0.34%	0.30%	0.11%	0.55%
4 weeks	**LFS**	0.32%	0.15%	0.26%	0.23%	0.08%	0.42%
	**DWP**	0.28%	0.13%	0.23%	0.20%	0.07%	0.37%
2 weeks	**LFS**	0.16%	0.07%	0.13%	0.11%	0.04%	0.21%
	**DWP**	0.14%	0.06%	0.11%	0.10%	0.04%	0.18%

Adjusting for informal care reduces the estimated cost of school closure to between £398 million to £453 million per week, as compared to our baseline estimate of ~£1 billion per week (Table 2). Adjusting for the elasticity of production, suggests that the aggregate cost of school closure reduces to £692–£788 million per week. The cost of productivity loss when adjusted by the proportion of workforce able to work from home reduces to £605–£690 million per week. When effective labour, informal care, and working from home are adjusted simultaneously, the productivity cost of school closure falls even further (in the range of £222 million to £254 million per week). If men are as likely to take absenteeism to care for dependent children as women, then the estimated cost of school closure is increased by about 30% (Table 2) of the base case estimate, as average male wages are higher. The cost as a % of GDP for the above adjustments is also reported in Table 3.

## Discussion

By no means all of the workforce would be affected by a policy of school closure. We estimate that about 16% of the workforce may take absenteeism because they are probably the main care-giver of dependent children. Nevertheless this level of absenteeism could have a significant impact on the economy. Our estimates suggest that the cost of school closure would be £0.2 bn to £1.2 bn per week. This amounts to around 0.2–1% of GDP for school closure lasting the duration of a pandemic wave (around 12 weeks). These estimates exclude the possible inflationary effects that could result from prolonged school closure, from the increasing costs to firms [25]. Although the motivation for estimating these costs is pandemic planning, they are not specific to an epidemic of influenza, and would arise from other sources of school closure (such as a national teacher's strike).

Our estimates are dependent on a number of assumptions and are subject to significant uncertainty, as shown by the scenario analysis. It is clear from this, that the proportion of individuals who may be able to access informal care is a critical determinant of the cost of a school-closure policy. However, it should also be remembered that the use of informal care over prolonged periods may be difficult to arrange, and could reduce the desired benefits of school closure, if children are kept in relatively large informal groups. A further assumption is that workers who are at home caring for their dependent children are unable to work. It is likely that many workers in certain sectors of the economy, would be able to do some work from home, at least on a part-time basis. We were not able to obtain specific estimates of what proportion of the workforce might be able to work from home, and what level of productivity would likely result from such arrangement. The most appropriate proxy measure we could find was the proportion of homes that have access to broadband (with the implicit assumption that those that do have access are able to work at full capacity). Our estimates of the impact of this are correspondingly speculative.

The health-care sector employs a high proportion of women, compared to other sectors of the economy, and home-working is likely to be virtually impossible for many staff. As a result, this sector is likely to be most badly affected by a policy of school closure. Our estimates suggest that about a third of the workforce in this sector have dependent children under 16 years in the home, and might therefore have to take time off to care for their children. This is absenteeism in the absence of illness. At the peak more than 10% of the workforce would be expected to be absent through pandemic influenza [9] and about 5% from other causes [26]. That is, school closure, plus illness absenteeism, could reduce the workforce of the health sector by 45% at the peak of the epidemic – up to a third through school closure (assuming no adjustments due to informal care arrangements etc), and around 15% of the remainder from illness. Such levels of absenteeism, coupled with the dramatic increase in demand for health services that would be expected during a pandemic, would put the remainder of the health-related work force under severe strain. Note also, that if our estimates are broadly applicable, then the health service will be severely stretched throughout the period of school closure, and that even at the peak of the epidemic the majority of absenteeism in this sector might result from school closure rather than staff illness.

There are a number of limitations of this study. These include significant uncertainty in a number of important parameters (as mentioned above). In addition, however, we have not included any possible long term cost of school closure, such as extra teaching efforts required to bring the children up to level, or the cost associated with later entry into the labour force, or the costs resulting from a poorer educated workforce. Such costs would be difficult to estimate, and by ignoring them out analysis will be conservative in this respect.

## Conclusion

Epidemiological [8] and modelling studies [5-7,9,10] have suggested that school closure can help in mitigating the effects of a pandemic, – particularly by reducing illness rates in children. This is the first study that we are aware of that has attempted to estimate the cost of such a policy. The estimates of school closure associated absenteeism and the projected cost would be useful for pandemic planning for business continuity, and for cost effectiveness evaluation of different pandemic influenza mitigation strategies. The potential benefits of school closure need to be weighed against the possible costs, to determine the best course of action. That is, although the costs of school closure, as estimated here, might appear to be large, the benefits of the policy, in terms of cases and deaths prevented and consequent savings to the health sector and society, might be acceptable. Only a full economic analysis can shed light on whether such a policy should be adopted.

## Competing interests

The authors declare that they have no competing interests.

## Authors' contributions

MZS performed the data analysis and drafted the manuscript. EJA extracted the data and helped drafting the paper. WJE analysed data and drafted the manuscript. All authors read and approved the final manuscript.

## Pre-publication history

The pre-publication history for this paper can be accessed here:


